# Current situation of vaccine injury compensation program and a future perspective in light of COVID-19 and emerging viral diseases

**DOI:** 10.12688/f1000research.51160.1

**Published:** 2021-07-26

**Authors:** Tommie Crum, Kirsten Mooney, Birendra R. Tiwari

**Affiliations:** 1 Department of Microbiology, Saint James School of Medicine, Albert Lake Dr The Quarter, AL-2640, Anguilla; 2 Department of Microbiology and Immunology, American University of Barbados, School of Medicine, Saint Michael, Wildey, BB 11100, Barbados

**Keywords:** Vaccine, Vaccine Injury Compensation Programs, Immunization, COVID-19 vaccines, emerging viral diseases

## Abstract

**Background:** Vaccines have had a great impact on disease prevention and mortality reduction. Very rarely, vaccines also can result in serious adverse effects. In consideration of this fact, vaccine injury compensation programs have been implemented in many countries to compensate a vaccinee for associated adverse effects. The existing vaccine injury compensation system addresses routine immunization schemes. However, there are rising concerns about the compensation for adverse effects caused by new vaccines such as those developed for coronavirus disease 2019 (COVID-19). The objective of this article is to review the existing vaccine injury compensation programs in different countries. The review also highlights the necessity to include all upcoming new vaccines for COVID-19 and other emerging viral diseases in the compensation schemes.

**Methods** **: **Published articles relating to vaccine compensation injury programs, vaccines, injuries, disabilities, illnesses, and deaths resulting from vaccination were searched in data bases. Through a careful review of the abstracts, 25 relevant articles were selected for analysis.

**Results**: We identified 27 countries on four continents with vaccine injury compensation schemes: 17 countries in Europe, 7 countries in Asia, the United States, a Canadian Province and New Zealand. No programs were identified in Africa and in South America. Program design, funding, and eligibility for compensation vary vastly between countries. We identified 17 countries operating well-established vaccine injury compensation programs. However, minimal information is available on numerous other countries.

**Conclusion**: We have identified 27 countries operating vaccine injury compensation programs. In Canada, Quebec is the only province with a scheme; however, discussions are ongoing in Canada for nationwide implementation in light of COVID 19.  Study limitations include limited scientific material, which hindered our research. Additional data concerning payout for each type of injury and the number of claimants related to a specific vaccine injury worldwide could provide a more comprehensive analysis.

## Introduction

Vaccines have been used for years to induce herd immunity and confer resistance to disease. Vaccines have had a great impact on health, economics and social welfare. ^1^ Second only to safe drinking water, vaccinations have improved on mortality reduction and are the most cost-effective health intervention. ^2^ Normally vaccines are licensed after years of safety testing processes. Initial vaccine testing is carried out on animal models and subsequent testing employs thousands of human participants. Multiple stage clinical trials assure that vaccines do not confer serious injury to most people. ^3^ However, likewise any medication, their side effects do pose a risk. The main components of vaccines are microbial antigens as active immunizing agents. Depending on the type of vaccine, the immunizing agent can be a microbial product such as a toxoid, microbial proteins, capsular components and whole live attenuated or killed organisms. In addition, vaccines are produced with a number of other substances such as aluminum hydroxide, mineral oils, gelatin, polyethylene glycol and antibiotics. These substances function as an adjuvant, stabilizer or preservative in the vaccine. Furthermore, vaccines may be contaminated with the residue of cell cultures if the organisms of interest were propagated in cell lines for antigen production. Therefore, in rare cases vaccines have shown to pose the possibility of causing mild to serious hypersensitivity reactions in individuals allergic to any of the constituents of the vaccine. ^4^ These rare adverse events can result in anaphylaxis, brachial neuritis, encephalopathy, thrombocytopenia, skin rashes, arthritis, Guillain-Barre syndrome, bowel intussusceptions and narcolepsy. ^5^^,^^6^

Germany was the first country to initiate a vaccine injury compensation program (VICP) in 1961 when the Supreme Court judged in favor of a vaccinee who was injured by the administration of a smallpox vaccine. The VICP was designed to be a no-fault claim system and were instituted as an alternative to the traditional civil law system for resolving vaccine injury claims. In a no-fault claim system, the claimant must prove the vaccination to be the cause of injury rather than the injury being at medical fault. ^7^^,^^8^ Although, originally the VICP aimed to mainly compensate vaccinees who sustained an injury, the VICP plays several roles in today’s society. The expanded role of the current VICP includes maintaining adequate supplies of vaccines at a reasonable cost, to protect physicians from liability, and to encourage pharmaceuticals companies to develop new vaccines. ^9^

Considerable amounts of compensation have been provided for vaccine injuries in various countries through their respective programs since the inception of VICPs. For example, the United States of America’s (USA) vaccine compensation program has awarded compensation to more than 6,276 families and individuals totaling to $4.7 billion since its inception in 1986. ^10^

VICPs are increasingly relevant in the present global situation given the current coronavirus 2019 (COVID-19) pandemic and the need to rapidly establish public trust in immunization. The world has been continually facing serious public health threats with several emerging and reemerging infectious diseases. ^11^ The COVID-19 pandemic, caused by a novel virus, severe acute respiratory syndrome coronavirus-2 (SARS-CoV-2), that began in December 2019 from Wuhan City, Hubei Province of China ^12^^,^^13^ is the most terrible scourge of humanity in over a century. A search of ongoing vaccines platforms for COVID-19 in ClinicalTrials.gov reveals approximately 223 candidate vaccines under development in different stages, 11 of them have reached to phase III clinical trials. Two candidate mRNA vaccines (developed by Pfizer/BioNTech, and Moderna) and two recombinant vaccines developed by Oxford-AstraZeneca and Johnson & Johnson have received emergency use authorization from the World Health Organization (WHO) and the Governments of several countries including USA, United Kingdom (UK), United Arab Emirates (UAE), European Union (EU) and India. In addition, CoronaVac of Sinopharm is an inactivated version of the SARS-CoV-2 has been authorized in China. Sputnik V, an adenovirus vector vaccine has been authorized in Russia. Vaccination is already in progress worldwide and as of June 16, 2021 over 2 billion and 777 million vaccine doses have been administered ( covid19.who.int). An enormous number of vaccine injury events are expected as vaccinations continue in the days ahead. In light of these circumstances, all of the COVID-19 vaccines which received the emergency use authorization have to be included in a compensation scheme. The new updated scheme should be well defined and equitable so that it can include other new vaccines which are under developmental stages targeting emerging and re-emerging viral diseases. ^14^

The objective of this article is to identify the current status of VICPs in multiple countries operating a  VICP, reporting specific vaccines that are covered, and identifying funding sources for their compensation program. This article will also highlight the need of vaccine injury compensation programs in light of the current COVID-19 pandemic and many other emerging diseases.

## Methods

We started developing a conceptual framework about vaccine injury compensation after discussion among the authors in April 2019; soon after, a literature search was run. We kept on searching and the last literature search was carried out on 16 June 2021. Based on the objectives, inclusion and exclusion criteria were set. We included countries with no-fault VICPs and peer-reviewed articles were reviewed regarding the VICPs as well as gray literature (law, guidelines, reports, and bulletins). We excluded publications of vaccine clinical trial-related injuries and compensations due to faulty medical practices. The main database searched was MEDLINE (PubMed interface) using medical subject heading ( *MeSH*) terms for relevant literatures. Additionally, relevant gray literature was retrieved by Google searching. Articles published from January 1979 to April 2021 were searched. Several relevant keywords and phrases such as ‘vaccine injury compensation program’, ‘no-fault compensations system’, ‘vaccine adverse effects’, ‘disabilities’, and ‘illnesses’ were used in the search string while searching through Google. Variables such as countries with VICPs, payout amount, funding source were used for the extraction of data. Since this is a qualitative, non-meta-analysis review, data were extracted manually and findings are presented in summary tables. Funding sources for injury compensation and operation of the VICP in different countries were identified from country specific literature searching. All three authors were involved in the review process independently, and the final selection was made with collective discussion among the authors.

## Results

**Figure 1.**
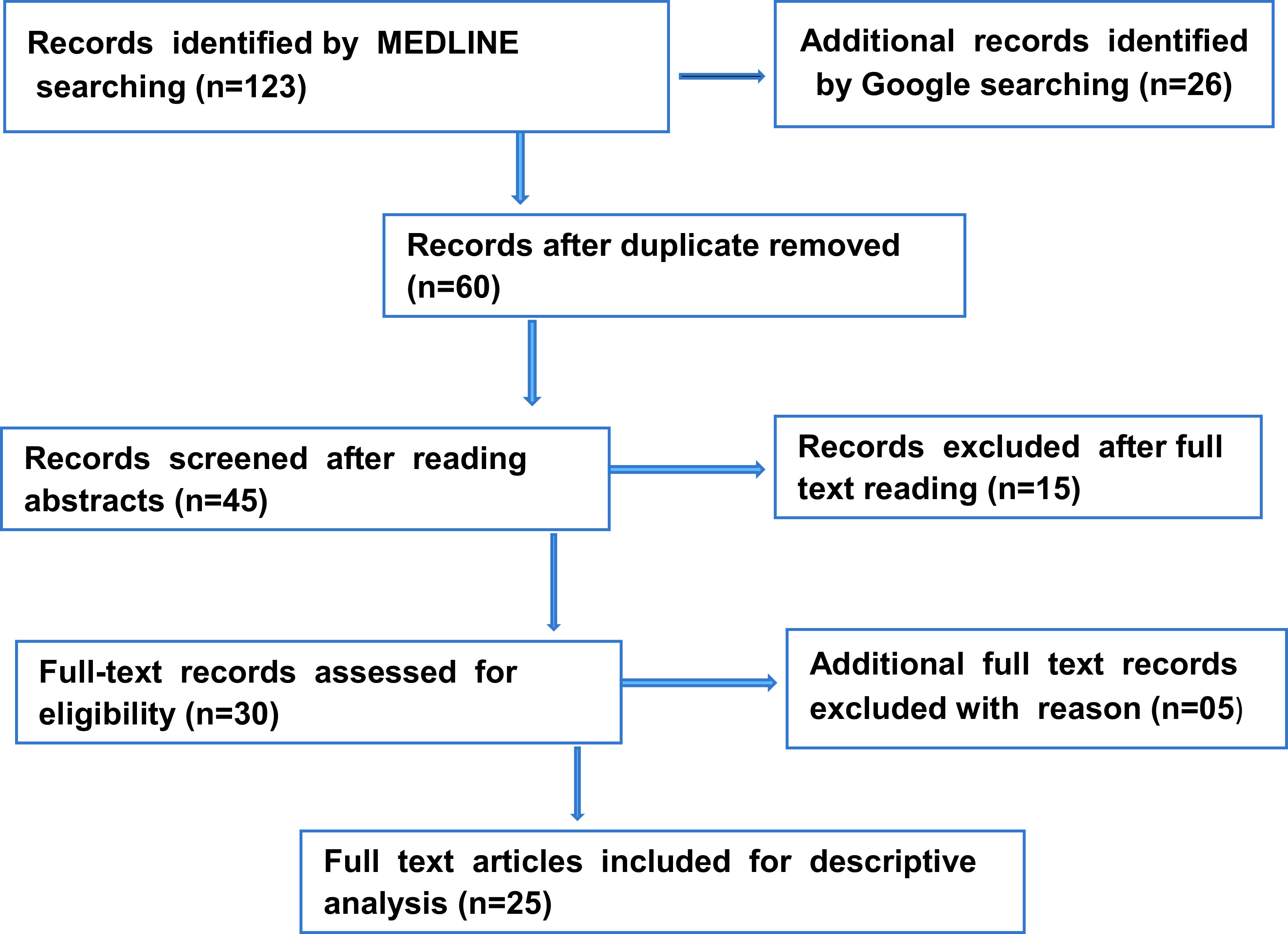
Flow chart reporting the number of records identified from database searching.

A total of 123 articles were retrieved by MEDLINE and another 26 were retrieved from Google searches. After assessing the articles, 25 articles were selected based on inclusion criteria for analysis in this study ( Figure 1).

## Current global situations of VICPs

Throughout researching VICPs we identified 27 countries ( Table 1) having VICPs on four continents. No VICP was identified in Africa or South America. Most of the developed countries have instituted and implemented adroitly rounded and evolving vaccine injury compensation programs. However, minimal information is available from several other countries. ^15^^-^^17^

**Table 1.** Countries and provinces those are operating a vaccine injury compensation program (VICP) in six continents.

Continents	Countries	References	Remarks
Europe	Denmark, Finland, Sweden, Switzerland, Germany, UK, Italy, Norway, France, Austria, Hungary, Iceland, Luxembourg, Norway, Russia, Latvia, Slovenia	15, 18- 28	Minimal information is available on Slovenia, Austria, Hungary, Iceland, Luxembourg, Russia, Latvia
Asia	Japan, China, South Korea, Taiwan, Nepal, Vietnam, Thailand	15, 29- 32	Minimal information is available on Nepal, Vietnam and Thailand
North America	Canada, USA	15	At this time the only province in Canada that provides compensation is Quebec
Australia	New Zealand	15	
Africa	No VICPs identified	16	All vaccine programs in Africa are administered through the Expanded Program on Immunization (EPI)
South America	No VICPs identified	17	No VICPs

**Table 2.** Pay out amount and funding sources in different countries with well instituted vaccine injury compensation programs.

Country	Pay out in US dollars	Funding source	References
**Denmark**	Up to$100,000	Central Taxation & Social Security	18, 19
**Finland**	$992,000	Pharmaceutical Injury Insurance	20
**Norway**	1.1 Million	Pharmaceutical Association	21
**Sweden**	$105,000	Pharmaceutical Injury Insurance Government Insurance	22, 23
**Switzerland**	No data available	Cantons, Federal Subsidies	24
**Germany**	No data available	General Revenue of the Lander (state governments)	25, 26
**France**	No data available	National Treasury	26
**United** **Kingdom**	$155,000	Public funds, National Treasury	26, 27
**Italy**	No data available	Ministry of Health	28
**South Korea**	Above $300	Korean National Vaccine Injury Compensation Program (KNVICP)	29, 30
**China**	$10,400	Government or by vaccine manufacturer	31
**Japan**	Up to 1 million	Class 1 - Health Service Bureau Class 2 - Pharmaceutical and Food Safety Bureau (PFSB)	32
**Taiwan**	Up to 200000	Through a 0.05 tax on each vaccine dose	33, 34
**Canada (Quebec)**	4.4 Million	Provincial Ministry of Health and Social Services	35, 36
**New Zealand**	$2,850 average	Government funding and investment returns	37
**USA**	4.7 billion to date	Through tax on each vaccine dose	39- 41

## Evaluation of VICPs by country

**Denmark** – Denmark’s VICP was first established in 1972. The most frequent vaccines that occur in compensation litigation in Denmark include pertussis, diphtheria, tetanus, polio and tuberculosis. The minimum financial compensation is $60 USD equivalent with the average payout of $100,000 USD equivalent. Denmark’s program is funded through central taxation and social security revenues. The Danish Patient Insurance Association is the governing body that confirms any injuries and awards any specific compensation. In Denmark, coverage is provided for the following: loss of earnings or loss of work capacity, permanent injury, pain and suffering, as well as health expenses relating to treatment. In case of death, compensation can also provide death benefits for dependent family members and for the coverage of funeral expenses. The standard of proof in Denmark requires more than 51% likelihood that the injury is associated with the vaccine. The people of Denmark enjoy overall satisfaction with their VICP. ^18, 19^

**Finland –** Finland’s VICP was first established in 1984. Finland maintains a cooperative that vaccine manufacturers are required to join to be covered in the case of litigation against them for vaccine injury compensation. Litigation against a vaccine manufacturer can include compensation for pain and suffering, permanent disability, cosmetic injuries, scars and narcolepsy. Finland only requires a 50% standard of proof to approve compensation. Finland boasts a high award rate for compensation programs at 50% of all filed claims receiving an award. Finland awards approximately $992,000 USD equivalent annually to claimants. Finland’s program is funded through the pharmaceutical injury insurance program. ^20^

**Norway** – Norway’s VICP was first established in 1995 and funded by the pharmaceutical association. The average yearly monetary payout provided by the Norwegian’s VICP for vaccination related injuries is $1.1 Million USD equivalent. Norwegians file less than 100 claims per year for vaccine related injuries. The Pharmaceutical Association reports a 40% success rate for a claim that is filed to receive compensation. In Norway, a 51% chance that the injury is caused by the vaccine in question is required for compensation to be rewarded. ^21^

**Sweden** – Sweden’s VICP was first established in 1978. Sweden provides approximately $105,000 USD equivalent per approved claim to no-fault injury claimants with a success rate of 35%. The pharmaceutical industry and Government insurance both provide financial coverage for compensation payouts. In Sweden, a proposed vaccine injury victim needs only to provide probable cause to file a petition. Approximately 626 claims are filed each year for vaccine injuries in Sweden. ^22^^,^^23^

**Switzerland** – Switzerland’s VICP was first established in 1970. Switzerland provides compensation for vaccine injuries related to mandatory or officially recommended vaccines and all pathologies subsequent to vaccinations excluding self-harm. There program is funded through Cantons Federal Subsidies. Individual cantons are responsible for developing their own procedures to implement federal vaccine compensation laws. A canton may deny compensation for a vaccine injury if compensation has been provided from another source. Cantons receive a federal subsidy for up to 25% up their operating expenses. The cantons in Switzerland are comparable to the states in the United States of America. ^24^

**Germany** – Germany’s VICP was first established in 1961. Germany’s VICP covers mandatory and recommended vaccinations which vary year by year. Coverage is awarded for not only death benefits but survivor benefits as well. Also included is compensation in the form of social assistance for health wellbeing and economic consequences of injuries. Germany’s VICP is funded through general revenue of the Länder (State Government). Germany’s program set the precedent for VICP establishments by becoming the first program in operation in 1961. ^25^^,^^26^

**France –** France’s VICP was first established in 1963. France’s VICP covers state required and mandatory vaccines. The program is administered through the Ministry of Solidarity and Health. Eligible claims include injuries only directly attributable to the vaccine. France states that the standard of proof must be convincing and clear evidence presented within 4 years post vaccine injury. Types of awards provided for compensation through France’s VICP include non-economic losses, pensions, disability, funeral and medical expenses. Financial loss of support for dependents of vaccine injury victims is also available through France’s program. France’s VICP is funded through the National Treasury. In France, an administrative tribunal makes choices pertaining to vaccine injury compensations. France averages approximately 39 vaccine injury claims per year. ^26^

**United Kingdom** – United Kingdom’s VICP was first established in 1978. The United Kingdom’s VICP is called the Vaccine Damages Payment Scheme (VDPS) which was created under the Vaccine Damages Payment Act in 1979. The VDPS is directed under the Department of Work and Pensions (Responsible for accessing claims for damages) as well as the Department of Health (Responsible for policy). The United Kingdom provides compensation for injuries resulting from the administration of recommended childhood vaccines and for adults who have been vaccinated with influenza, diphtheria, tetanus, influenza, poliomyelitis, measles, rubella, tuberculosis, and smallpox. Armed forces vaccinations are also covered under the adult vaccine compensation program. A unique aspect of the United Kingdom’s VICP is that any person making a fraudulent claim is liable for a $1,285 USD equivalent fine. The United Kingdom’s VICP is funded through the National Treasury. The United Kingdom’s compensation program pays out an average of $2 million USD equivalent per year to approved claimants. Filing must occur within 6 years post injury. The maximum award paid out per approved claim is $155,000 USD equivalent. ^26^^,^^27^

**Italy** – Italy’s VICP was first established in 1992. Italy covers vaccine injuries through their VICP for compensation of state recommended vaccines including diphtheria, tetanus, polio, hepatitis B, measles, mumps, rubella (MMR), human papilloma virus (HPV),rotavirus, and pertussis. Claimants can seek compensation including benefits for medical expenses, death benefits, Kawasaki disease, Henoch Schonlein Purpura, allergic reactions, thrombocytopenia, and hemolytic anemia. The VICP in Italy is funded through the Ministry of Health of the Italian Government. ^28^

**South Korea** – Korea’s VICP was first established in 1994. Korea covers vaccine injuries resulting from vaccination with diphtheria, pertussis, tetanus (DtaP), MMR, Bacille Calmette Guerin (BCG), Japanese encephalitis, Korean hemorrhagic fever, and influenza. Korea’s program is based on the WHO causality assessment criteria. The Korean National Vaccine Injury Compensation Program (KNVICP) provides compensation for victims of vaccine injury. Almost 68% of vaccine injury claims are successfully compensated in Korea. Korea requires that a claimant has spent more than $300 USD equivalent on medical expenses. ^29^^,^^30^

**China** – China’s VICP was first established in 1988. In China, the oral polio, measles, hepatitis B vaccine (HBV) and meningococcal polysaccharide vaccine (MPV) vaccines are all covered for compensation injuries limited to death and disability only. The average pay out per approved claimant is $10,400 USD equivalent. Funding per payout compensation is provided by the government and the vaccine manufacturer. China’s complicated and laborious process of filing for vaccine compensation includes a three-stage process which results in a very timely and expensive process for claimants. Epidemiological studies are required to prove that the vaccine in question resulted in the injury sustained. Public protest has occurred on numerous occasions due to the masses being profoundly disappointed in the system of vaccine injury adjudication in China. ^31^

**Japan** – Japan’s VICP was first established in 1970. Japan provides compensation for diphtheria, poliomyelitis, measles, mumps, rubella, Japanese B encephalitis, tetanus, tuberculosis and smallpox vaccine (class I). Influenza vaccine (class II). Compensations include health care benefits, medical allowances, funeral, death, disability, and injury coverage. Compensation amount is paid either through the Health Service Bureau or through the Pharmaceutical and Food Safety Bureau (PFSB). Japan has established a set amount per pay out, if the injury has an undisputed correlation with the vaccine. The maximum awarded compensation is $1 Million USD equivalent. Due to the stringent requirements for filing and limitations placed upon claimants, very few cases are advanced into the court system. The resulting vaccine award rate per claim is 80%. ^32^

**Taiwan** – Taiwan’s VICP, the Vaccine Injury Compensation Program Working Group (VICPWG), was first established in 1988. BCG, influenza and MMR are all covered through Taiwan’s VICP. The MMR vaccine makes up 33% of the payouts and the BCG vaccine is the most compensated vaccine. Injuries that receive compensation in Taiwan include physical and mental effects, serious injuries and death. These guidelines are similar to the World Health Organization’s recommendations for vaccine injury compensation programs, 98 vaccine injury compensation claims are paid out on average per year in Taiwan. This equates to a 40% success rate per claimant. Taiwan set the maximum payout amount for each injury at $200,000 USD equivalent. They provide compensation for physical and mental impairments at $165,000 USD equivalent per occurrence and for serious illness provides $33,000 USD equivalent. Mild adverse reactions receive a payment of only $6,500 USD equivalent per approved claim. In Taiwan, the Government provides funding for the VICPWG through a $0.05 USD equivalent tax on each vaccine dose that is purchased. ^33^^,^^34^

**Canada** – Canada’s VICP was first established in 1985. Canada maintains the position as the only country in the world to operate a VICP within their country that is not accessible toall citizens of the country. Currently, Quebec is the only province in Canada that provides vaccine compensation. Quebec develops, maintains, and funds their own vaccine compensation injury program for the citizens; in the event of a vaccine injury the remaining citizens of Canada are not eligible for participation in the program. In the province of Quebec, a citizen who is injured by administration of a vaccination must provide unequivocal proof of permanent impairment to be eligible to receive compensation for a vaccine injury. Their program has awarded an average of $4.4 Million USD equivalent through the Provincial Ministry of Health and Social Services. Vaccines that are covered are numerous and change regularly. ^35, 36^ According to the recent news, Canada announced a nationwide vaccine compensation injury program specifically designed to encourage confidence in COVID-19 vaccinations ( ctvnews.ca). This program will be operated separately from Quebec’s program and will only include coverage for the COVID-19 vaccinations. Minimal information has been released at this point, as it is still in its development.

**New Zealand** – New Zealand’s VICP was first established in 1974. New Zealand maintains a no-fault program with significant relative ease of approval of compensation due to a claimant’s lack of need to prove negligence by medical professionals. Vaccine injuries are uniquely identified as medical injuries by New Zealand’s VICP. New Zealand is able to maintain their VICP cost at an acceptable level through the program being intertwined with social and employment resources. New Zealand provides buy in opportunities for physicians, no legal fees for compensation seekers and caps on monetary compensation for claimants. New Zealand’s VICP requires a notification of injury within 12 months post injury and only permanent disability is covered for vaccine injury compensation. The average individual payout is $2,850 USD equivalent per claim, government funding supplement vaccine injury compensation programs in New Zealand. ^37^

**United State of America (USA)** – The USA’s VICP program was first established in 1988. In the USA recommended childhood vaccines and adult influenza vaccine recovered under the National VICP. Compensable injuries vary per vaccine. For DTaP, MMR, and HBV, injuries including anaphylaxis, brachial neuritis, arthritis and thrombocytopenic purpura are compensated. For all covered vaccines both permanent disability and death are compensated. In the USA, a 3-year filing deadline is implemented. The government maintains a vaccine injury table and follows it for proof of causality for their standard of proof. Off table claims do occur and special groups of the VICP are assigned to handle all off table claims and adjudication. These claimants are required to provide undeniably high levels of epidemiological evidence for their claim. These claimants usually require legal representation to handle the immensity of their claims. Covered compensation would include medical bills, lost wages, future care costs, attorney’s fees, death and non-economic losses. The USA levies a tax on every vaccine dose that is distributed to cover the cost of the program. The USA provides a vaccine court to adjunct claims. However, ultimately 80% of cases are negotiated and settled out of court. The USA’s VICP has an average of 643 claims per year with 34% of those claims receiving compensation. Approximately, $4.7 billion dollars has been paid out to vaccine injury victims through the vaccine courts in the USA to date. ^38^^-^^41^

## Future perspective of VICPs in light of COVID-19 and other emerging diseases

Although, enormous strides have been made in the areas of compensating families affected by vaccine injuries, the world’s governments have concurred that improvements in these programs should continue to be developed. Development and expansion of vaccine injury compensation programs would continue to encourage responsible medicine as well as thoughtful humanitarianism worldwide. The existing no-fault vaccine-injury compensation schemes in 27 countries are for routine immunizations; however, these systems are not always well defined to cover the losses or damages of vaccine administration during pandemic crises. The presently ongoing COVID-19 pandemic devastation urges today’s society to redefine and employ the VICPs in an urgent manner. An improved VICP would ensure a competent and productive program to match the needs of today’s ever evolving world situation and to provide safe and equitable access to vaccines. It is likely that over a billion doses of COVID-19 vaccines will be administered worldwide by the end of 2021.The recently launched COVID-19 mRNA vaccines were developed by using a very new platform. This technology utilizes the mRNA complexed with cationic lipid nanoparticles, cholesterol and polyethylene glycol. ^42^ Emergency use authorization has been granted because of the urgent need. Therefore, phase III clinical trials of these vaccines had a limited sample size and duration. Some serious vaccine related adverse effects can be expected after the administration of new vaccine for the first time in humans. ^43^ Severe adverse reactions including anaphylaxis and blood clotting disorders have been reported after administration of COVID-19 vaccines. ^44^^,^^45^ In addition to COVID-19, presently, there are several ongoing emerging and reemerging viral diseases pandemic in nature and several candidate vaccines platforms are in the pipelines to respond these diseases. ^46^ As more vaccines are made available for human use, more vaccine injury events can be expected. Considering the current situations, there are suggestions for the revision of the existing vaccine injury compensation system. ^47^ A few already practicing mechanisms can be adapted, such as the World Health Organization (WHO) insurance system for vaccines during a public health emergency initiative COVID-19 Vaccines Global Access (COVAX). The COVAX authority can be used to promote improvements in regional or national compensation system for member countries. One example of a possible improvement is for all programs to add a nominal per-dose tax to the manufacturer. ^47^

## Conclusion

VICPs are established on the principle of no-fault claims. This principle states that the adverse effects are not related to an individual’s fault or a vaccine manufacturer’s misdeed; they are inevitable consequence of medical interventions. There is no standard definition for causation. Eligibility criteria for compensation are often based on WHO guidelines or the specific country’s operating principles and decisions. The lowest vaccine injury payout was identified in New Zealand and the highest was identified in Norway. The USA’s program is the costliest due to its legal based structure. The VICPs process also supports supply of vaccines, maintains the cost, and protects physicians from liability. Compensation programs have increasingly restored public confidence in the safety of vaccinations and their modifications to adjust to current world issues. Additional studies as new information is available may provide comprehensive data on individual countries enhancing our overall research. Additional data concerning payout for each type of injury and number of claimants of all VICPs worldwide could also provide a more comprehensive analysis. Further research would be advantageous in respect to other countries currently having minimal information about VICPs. Billions of people are expected to receive new vaccines for COVID-19 by the end of 2021; there are valid and founded concerns to initiate VICP modifications worldwide to accommodate the changing situation. VICPs must be redefined to include several upcoming vaccine platforms for emergency uses targeting emerging and reemerging diseases.

## Data availability

All data underlying the results are available as part of the article and no additional source data are required.
