# Supplementary material for: Current situation of vaccine injury compensation program and a future perspective in light of COVID-19 and emerging viral diseases
Source: F1000Res. 2021 Dec 7;10:652. Originally published 2021 Jul 26. [Version 2] doi: 10.12688/f1000research.51160.2 (PMC8733825; doi:10.12688/f1000research.51160.2)
Supplement: Peer review history

# Peer review: Reviewer response for version 2

**DOI:** 10.5256/f1000research.79834.r102151

**Published:** 2022-01-05

**Authors:** Lal Rawal

**Affiliations:** 1 School of Social Sciences and Psychology, Western Sydney University, Sydney, NSW, Australia

Authors have addressed my review comments/feedback provided earlier. I am happy to approve this revised version of the manuscript.

Is the work clearly and accurately presented and does it cite the current literature?

Yes

If applicable, is the statistical analysis and its interpretation appropriate?

Not applicable

Are all the source data underlying the results available to ensure full reproducibility?

Partly

Is the study design appropriate and is the work technically sound?

Partly

Are the conclusions drawn adequately supported by the results?

Partly

Are sufficient details of methods and analysis provided to allow replication by others?

Partly

Reviewer Expertise:

NA

I confirm that I have read this submission and believe that I have an appropriate level of expertise to confirm that it is of an acceptable scientific standard.

# Peer review: Reviewer response for version 1

**DOI:** 10.5256/f1000research.54292.r90430

**Published:** 2021-10-19

**Authors:** Keshab Parajuli

**Affiliations:** 1 Department of Clinical Microbiology, Institute of Medicine, Tribhuvan University Teaching Hospital, Kathmandu, Nepal

- This review article entitled “Current situation of vaccine injury compensation program and a future perspective in light of covid-19 and emerging viral diseases” is well written and has included very relevant information regarding the objectives.
- Since there are a lot of ambiguities regarding no-fault compensation schemes, this article provides comprehensive details for future researchers. I found sufficient details in the methods and appropriately interpreted.
- Conclusions are drawn appropriately supporting and representing the whole article in a glance.

I  approve this review for indexing.

Is the work clearly and accurately presented and does it cite the current literature?

Yes

If applicable, is the statistical analysis and its interpretation appropriate?

Not applicable

Are all the source data underlying the results available to ensure full reproducibility?

Yes

Is the study design appropriate and is the work technically sound?

Yes

Are the conclusions drawn adequately supported by the results?

Yes

Are sufficient details of methods and analysis provided to allow replication by others?

Yes

Reviewer Expertise:

Microbiology immunology

I confirm that I have read this submission and believe that I have an appropriate level of expertise to confirm that it is of an acceptable scientific standard.

# Peer review: Reviewer response for version 1

**DOI:** 10.5256/f1000research.54292.r93932

**Published:** 2021-10-08

**Authors:** Lal Rawal

**Affiliations:** 1 School of Social Sciences and Psychology, Western Sydney University, Sydney, NSW, Australia

Overall, this research work has a great public health importance and thanks to the authors for such great works. Followings are some of my observations and comments/ feedback. I hope these will help authors to improve the quality of the paper.

**Abstract:**

- Abstract, results: Please clearly state the information relating to continents and countries, where the studies were conducted.
- How did authors assess/ determine the well-established program in 17 countries? It's unclear. I invite authors to clearly state these.
- Abstract: The conclusions in the abstract sub-section do not align with the findings presented above. I invite authors to reframe it. Perhaps like: We conclude that the vaccine injury prevention and compensation programs are available limited number of countries across four continents - mostly in Europe. Lack of standard approach and scope of injury prevention and compensation programs across the countries exists. Some important limitations include: 'Therefore, additional data...'

**Body of the manuscript, **

- Page 3, methods section:
  - The methods section needs standard/ more academic way of presenting the work, perhaps like: Using a standard framework to review the literature, we searched key online databases for relevant literature published till 16 June 2021.
  - Did you use a review framework like PRISMA or STROBE guidelines? If so, you could then include it here.
- Page 4, methods, para 1: The dates of literature search and reviewed mentioned in the page 4 para 1 is inconsistent with the ones noted in page 3, last para. These dates need maintaining consistency.
- I have added some minor observations in the manuscript. Please find the manuscript with my edits/ comments attached here.
- Page 4, results section: Authors have noted 25 articles, but the programs have been available in 27 countries. If this is correct, I would invite authors to clearly state these details.
- Page 8, conclusion section: This conclusion section needs re framing. Please see the comments/ provided in the Abstract section. I invite authors to reframe it with developing conclusions based on the findings presented above, followed by some specific and doable recommendations.

Is the work clearly and accurately presented and does it cite the current literature?

Yes

If applicable, is the statistical analysis and its interpretation appropriate?

Not applicable

Are all the source data underlying the results available to ensure full reproducibility?

Partly

Is the study design appropriate and is the work technically sound?

Partly

Are the conclusions drawn adequately supported by the results?

Partly

Are sufficient details of methods and analysis provided to allow replication by others?

Partly

Reviewer Expertise:

NA

I confirm that I have read this submission and believe that I have an appropriate level of expertise to confirm that it is of an acceptable scientific standard, however I have significant reservations, as outlined above.

# Response

**Published:** 2021-11-26

**Authors:** Birendra Tiwari

**Affiliations:** American University of Barbados, School of Medicine, Barbados

Dear Reviewer, 

 Thank you for your time and valuable comments. I have revised the manuscript addressing your comments and suggestions. The clarifications of few of your queries have been mentioned below.

- *Abstract, Results: Please clearly state the information relating to continents and countries, where the studies were conducted.*
- **Response: **Given on the table 1 and 2, because of the word limit in the abstract we could not mention all the countries and continents here. 
- *Authors have noted 25 articles, but the programs have been available in 27 countries. If this is correct, I would invite authors to clearly state these details.*
- **Response: **Few records have included the VICP of 2 or more countries. For example, the reference 26 provide the information of 3 countries (Germany, France and United Kingdom)
